# Predicting acute lung injury in infants with congenital heart disease after cardiopulmonary bypass by gut microbiota

**DOI:** 10.3389/fimmu.2024.1362040

**Published:** 2024-10-24

**Authors:** Lan Jiang, Yueshuang Cun, Qiang Wang, Kede Wu, Menglong Hu, Zhen Wu, Tianyi Zhu, Zhaocong Yang, Nishant Patel, Xinyu Cai, Jirong Qi, Xuming Mo

**Affiliations:** ^1^ Department of Cardiothoracic Surgery, Children’s Hospital of Nanjing Medical University, Nanjing, China; ^2^ School of Public Health, Nanjing Medical University, Nanjing, China

**Keywords:** acute lung injury, congenital heart disease, cardiopulmonary bypass surgery, gut microbiota, biomarker

## Abstract

**Background:**

Acute lung injury (ALI) is a serious and common complication that occurs in children with congenital heart disease after cardiopulmonary bypass (CPB) surgery, leading to higher mortality rates and poorer prognosis. Currently, there is no reliable predictive strategy for CPB-associated lung injury (CPB-ALI) in infants. Certain characteristics of the gut microbiota could potentially serve as biomarkers for predicting the development of CPB-ALI.

**Methods:**

We conducted 16S rRNA sequencing to analyze the characteristics of the intestinal microbiota in healthy controls and infants with CHD admitted to the hospital. The CHD infants were divided into CPB-ALI and non-ALI (CPB-NALI) groups based on postoperative outcomes. Bacterial functional pathway prediction analysis was performed using PIRCUSt2, and the gut microbiota composition associated with immune status was determined with heatmap. Random forest regression models and ROC curves were utilized to predict the occurrence of CPB-ALI.

**Results:**

Our study revealed significantly different microbiota compositions among three groups (CON, CPB-ALI, and CPB-NALI). The microbiota diversity was low in the CPB-ALI group with high pathogen abundance and significant decrease in Bacteroides, while the opposite was observed in the CPB-NALI group. The microbiota dysbiosis index was high in the CPB-ALI group, with its dominant microbiota significantly associated with multiple metabolic pathways. Additionally, CPB-ALI patients showed high levels of inflammatory cytokines IL-8 and HMGB1 in their serum, with high expression of IL-8 being associated with Enterobacteriaceae. Further correlation analysis showed that the differences in gut bacterial taxonomy were related to the occurrence of ALI, length of stay in the cardiac care unit, and ventilation time. It is noteworthy that *Escherichia Shigella* performed best in distinguishing CPB-ALI patients from non-ALI patients.

**Conclusions:**

Our study suggests that postoperative ALI patients have distinct gut microbiota upon admission compared to non-ALI patients after surgery. Dysbiosis of the gut microbiota may potentially impact the progression of ALI through metabolic pathways, quorum sensing, and the levels of inflammatory factors expressed in the serum. *Escherichia Shigella* represents a potential predictive factor for the occurrence of ALI in CHD infants after surgery. Acute lung injury, congenital heart disease, cardiopulmonary bypass surgery, gut microbiota, biomarker

## 1 Introduction

Infants with complex congenital heart disease (CHD) often require surgical intervention using cardiopulmonary bypass (CPB) (1, 2), which can result in acute lung injury (ALI). ALI following CPB surgery is especially severe in infants and young children, with an incidence rate ranging from 15-60%. The prognosis is particularly poor in patients with cyanotic complex CHD, with mortality rates as high as 80% (3, 4). This complication has become a leading cause of increased mortality rates after cardiac surgery, imposing significant medical and social burdens (5, 6). Therefore, the identification of predictive biomarkers for CPB-related ALI in infants undergoing cardiac surgery is crucial for early diagnosis and effective treatment decisions.

During embryonic development, the lungs and intestines originate from the same foregut organ, leading to physiological similarities in their structure. In 2000, K R Cooke first proposed the concept of the “gut-lung axis,” highlighting the bidirectional connection between these two organs and the impact of dysbiosis in the gut ecosystem on lung infections, and vice versa (7). Intestinal ischemia-reperfusion injury is considered the initiating factor for CPB-induced ALI (CPB-ALI). It damages the intestinal epithelial cells, disrupts the natural barrier function of the intestine, and causes systemic endotoxemia, resulting in increased susceptibility to infections in other organs, particularly the lungs. Moreover, studies have demonstrated that the metabolic consequences of ischemia-reperfusion in the intestine contribute to the development of ALI. Numerous research studies have also reported a close correlation between the intestine and various types of ALI, such as trauma, burns, and sepsis. The intestinal microbiota and its metabolites play a critical role in regulating the gut-lung axis (8), the functional composition of the gut microbiota should be systematically studied in patients with ALI, especially the composition of the immune-related gut microbiota.

Previous studies have observed a positive correlation between post-CPB serum succinate and procalcitonin levels and the duration of mechanical ventilation, as well as a negative correlation with the oxygenation index. This suggests a link between lung injury after CPB and intestinal ischemia-reperfusion injury, highlighting the role of the microbial metabolite succinate in intestinal microbiota and its impact on lung injury, alveolar injury, and pulmonary inflammation following intestinal ischemia-reperfusion (9). Similar findings have been reported in adult patients undergoing CPB surgery, where an increase in pathogenic bacteria and a significant reduction in beneficial anaerobic intestinal bacteria has been observed, leading to an imbalance in the intestinal microbiota (10). Furthermore, children with congenital heart disease also experience dysbiosis and impaired intestinal barrier function after CPB, indicating a relationship between imbalanced intestinal microbiota during the perioperative period and the occurrence of lung injury following CPB (11).

The gut microbiota in infants exhibits unique characteristics and notable differences compared to the adult gut system (12–14). However, it remains unclear whether the gut microbiota in infants undergoing cardiac surgery is involved in CPB-ALI. Therefore, investigating the role of dysbiosis in CPB-ALI will not only enhance our understanding of the “gut microbiota-lung axis in infants” but also provide insights into potential therapeutic interventions. In this study, we examined the intestinal microbiota profiles of children with CHD upon admission and identified significant differences in the gut microbiota between infants with CPB-ALI and CPB-NALI (non-ALI induced by CPB). Our objective was to shed light on the contribution of gut microbiota composition to the development of these patients and explore potential predictive biomarkers of CPB-ALI in infants.

## 2 Materials and methods

### 2.1 Study design, population and sample collection

We conducted a prospective observational, single-center, 1:1 matched case-control preliminary study at the Children’s Hospital of Nanjing Medical University. The study received approval from the Ethics Committee of the Children’s Hospital of Nanjing Medical University (Identifier: 201912254-1), and informed consent was obtained from each enrolled patient or their legal guardian. We prospectively enrolled all children with congenital heart disease (CHD) aged ≤6 months who were admitted to the ICU and underwent cardiopulmonary bypass (CPB) surgery between May 2020 and October 2021. The following exclusion criteria were applied: 1) Cardiac surgery without CPB; 2) Age >6 months at the time of admission; 3) Cardiomyopathy; 4) Congenital renal anomalies, airway anomalies, or gastrointestinal anomalies; 5) Inherited metabolic diseases and chromosomal abnormalities; 6) Postoperative cardiogenic pulmonary edema or need for postoperative extracorporeal membrane oxygenation (ECMO); 7) A history of illness in the past month; 8) Receipt of complementary foods. For the control group, we enrolled the infants aged ≤6 months and excluded infants with congenital diseases or a history of illness in the past month to account for the potential influence of medication or antibiotics on the gut microbiota, their fecal samples were collected during hospital check-ups. Clinical characteristics were recorded according to standard procedures.

Fecal samples were collected from the CHD patients on the day of admission using disposable sterile rectal swab. The swabs were placed into test tubes containing RNAlater (Invitrogen, Vilnius, Lithuania), an RNA stabilization solution, in a volume ratio of 1:5-10 (RNAlater to sample) (15). Venous blood was collected from all CHD patients at 9–10 am on an empty stomach (at least 12 h fasting), mixed thoroughly and left to stand for 1 h, centrifuged at 4°C, 3000 rpm/min for 15 min. An equal volume of serum samples was aspirated into 1.5 mL EP tubes. All samples were stored at -80°C until further processing.

### 2.2 Clinical data

Clinical data including age, weight, gender, laboratory results, complications, and outcomes were extracted from medical records. Patients were classified into ALI (acute lung injury) and non-ALI groups based on whether the lowest PaO_2_/FiO_2_ ratio after corrective surgery was <100 mmHg. The diagnostic criteria for ALI were as follows: PaO_2_/FiO_2_ levels >200 mmHg ~ ≤300 mmHg was mild ALI; Moderate ALI was defined as >100 mmHg ~ ≤200 mmHg PaO_2_/FiO_2_ (16). Severe ALI was defined as ≤100 mmHg PaO_2_/FiO_2_ levels. The RACHS-1 score was used for risk adjustment in congenital heart surgery (17). Left ventricular ejection fraction (LVEF), pulmonary arterial pressure (PAH), and oxygen saturation (SPO_2_) were recorded. Preoperative white blood cell count (PreWBC), hemoglobin content (PreHb), platelet count (PrePlt), neutrophil count (PreN), lymphocyte count (PreL), and monocyte count (PreM), as well as the neutrophil-to-lymphocyte ratio (PreNL), were measured. CPB time, aortic cross-clamping (ACC) time, and the postoperative variables of partial pressure of arterial blood oxygen to inspired oxygen fraction ratio (PostPaO_2_/FiO_2_), white blood cell count (PostWBC), platelet count (PostPlt), neutrophil count (PostN), lymphocyte count (PostL), monocyte count (PostM), and neutrophil-to-lymphocyte ratio (PostNL) were investigated on the first postoperative day. Ventilator time and duration of stay in the cardiac care unit (CCU) were chosen as clinical outcomes.

### 2.3 Gut microbiota profiling

Microbial community genomic DNA was extracted from fecal samples using the E.Z.N.A.^®^ soil DNA Kit (Omega Bio-tek, Norcross, GA, U.S.) following the manufacturer’s instructions. The DNA extract was assessed on a 1% agarose gel, and DNA concentration and purity were determined using a NanoDrop 2000 UV-vis spectrophotometer (Thermo Scientific, Wilmington, USA). The V3-V4 hypervariable region of the bacterial 16S rRNA gene was amplified using primer pairs 343F (5’-TACGGRAGGCAGCAG-3’) and 798R (5’-AGGGTATCTAATCCT-3’) with an ABI GeneAmp^®^ 9700 PCR thermocycler (ABI, CA, USA). The 16S rRNA gene was amplified using PCR by first heating it to 95°C for 3 min. Then, the process included 27 cycles of heating the gene to 95°C for 30 s, cooling it to 55°C for 30 s, and extending it at 72°C for 45 s. Finally, there was a final extension at 72°C for 10 min before cooling the sample to 4°C. The PCR mixtures included 4 μL of 5 × TransStart FastPfu buffer, 2 μL of 2.5 mM dNTPs, 0.8 μL of 5 μM forward primer, 0.8 μL of 5 μM reverse primer, 0.4 μL of TransStart FastPfu DNA Polymerase, 10 ng of template DNA, and sufficient ddH_2_O to make a total volume of 20 μL. The PCR reactions were conducted three times as a measure of accuracy. The amplified PCR product was isolated from a gel made of 2% agarose, purified using the AxyPrep DNA Gel Extraction Kit (Axygen Biosciences, Union City, CA, USA). The purification process followed the instructions provided by the manufacturer. The resulting purified PCR product was then measured for its concentration using a Quantus™ Fluorometer (Promega, USA). The collected amplified DNA samples were combined in equal amounts and analyzed using paired-end sequencing on a NovaSeq PE250 platform (Illumina, San Diego, USA) by Majorbio Bio-Pharm Technology Co. Ltd. (Shanghai, China).

### 2.4 Analyzing and visualization sequencing data

All The data were analyzed on the online platform of Majorbio Cloud Platform (www.majorbio.com) (18). The UPARSE software (19) (version 11) was used to cluster sequences into operational taxonomic units (OTUs) at a 97% similarity threshold. The RDP classifier (20) (version 2.13) was then employed to assign taxonomic annotations to OTUs by aligning the sequences against the Silva 16S rRNA gene database (v138), using a confidence threshold of 70%. The composition of each sample’s community was subsequently assessed at different taxonomic levels. Functional prediction analysis of the 16S genes was performed using PICRUSt2 (21) (version 2.2.0).

#### 2.4.1 Alpha and beta diversity analysis

Alpha diversity metrics such as the observed richness (Sobs) and Shannon index were calculated using Mothur software (22) (http://www.mothur.org/wiki/Calculators). Inter-group differences in Alpha diversity were analyzed using Wilcoxon rank-sum test. PCoA analysis based on the Weighted UniFrac distance algorithm was used to investigate the similarity in microbial community structure among samples. PERMANOVA non-parametric test, in combination with PCoA, was employed to determine the significance of inter-group differences in microbial community structure. To determine the degree of microbial healthy status or dysbiosis, the Gut Microbiome Health Index (GMHI) and Microbial Dysbiosis Index (MDI) were evaluated. LEfSe analysis (Linear Discriminant Analysis Effect Size) (23) (LDA > 4, *p* < 0.05) was performed to identify bacterial taxa exhibiting significant differences in abundance from the genus to species levels between different groups. Additionally, a correlation network graph (24) was constructed using Spearman’s correlation analysis, with species selected based on |r| > 0.6 and *p* < 0.05.

#### 2.4.2 Anticipation of gut microbiota characteristic

The sequences were categorized using the GreenGenes database by running the USEARCH-otutab command (25). After that, the Bugbase tool (https://bugbase.cs.umn.edu/index.html) (26) was utilized to analyze the sequence library and forecast the characteristics of the gut microbiota. To conduct pathway prediction analysis, we utilized PICRUSt2 to forecast the potential functional makeup using the GO and KEGG databases (21). The microbial community’s predicted pathways were statistically compared using STAMP software (v2.1.3) and the Welch’s t-test.

#### 2.4.3 Random forest model

We divided the samples into a training set and testing set randomly, using 16S sequence profiles and clinical parameters. We employed the randomForest R package (27) to build a random forest regression model on 70% of the samples for the training set and reserved the remaining 30% for the testing set. The training set underwent tenfold cross-validation. We chose the best combination of microbial factors that had the least cross-validation error to forecast the occurrence of CPB-ALI. The most significant variables were utilized to create a predictive model, which was then assessed using ROC calculation to differentiate between patients with ALI and those without. The confidence intervals for the ROC curves were computed using the pROC R package (28). MedCalc software (v22.014) was used for statistical comparison of multiple predictive models (DeLong) (29).

### 2.5 Enzyme-linked immunosorbent assay

Peripheral whole blood of subjects in the fasting state was extracted using a collection vessel containing separation glue. After collecting the required specimens, the samples were gently reversed and mixed 3–5 times immediately. After the blood was completely coagulated, the samples were left at room temperature for 3500 r/min, centrifuged for 5 min, and the upper serum was absorbed. Human Elisa Kit (YOBIBIO, Shanghai, China) was used to detect the concentration of target protein in the serum of the subjects (IL-6, IL-8, TNF-a and HMGB1), and the absorbance OD value at 450 nm was detected using the kit and the DENLEY DRAGON Wellscan MK 3 enzyme-labeled instrument (Thermo, Finland), and the concentration of standard substance was taken as the horizontal coordinate. The OD value is the ordinate coordinate to draw the standard curve, and finally, the target protein concentration is calculated according to the OD value of the sample. To detect significant differences between two independent groups, either a Student’s t-test or Wilcoxon’s rank sum test was employed. All *p* values were two-sided, with a statistical significance threshold assumed for *p* < 0.05.

## 3 Results

### 3.1 The clinical features of patients enrolled in the study

The study consisted of 26 CHD patients with CPB, as well as 13 healthy individuals serving as controls. The CHD infants after CPB were categorized into CPB-ALI and CPB-NALI groups based on PaO_2_/FiO_2_ evaluation. The patient characteristics are summarized in **Table 1**. There were no significant differences between the CPB-ALI and CPB-NALI groups regarding age, gender, weight, breastfeeding, LVEF, PAH, SPO2, and RACHS-1 score (*p*>0.05). Additionally, there were no differences in laboratory parameters and surgical factors such as CPB time and ACC time between the two groups, except for preL (*p*=0.048). These results indicate that the impact of surgery and individual characteristics had a limited impact on the gut microbiota compositions and on clinical outcomes. PostPaO_2_/FiO_2_ (median 1.06, IQR 0.78-1.32 vs. median 2.80, IQR 2.38-3.25, *p*<0.001), postM (median 1.49, IQR 0.99-1.97 vs. median 0.85, IQR 0.57-1.11, *p*=0.005), and PostNL (median 5.66, IQR 4.35-8.06 vs. median 3.42, IQR 1.49-6.21, *p*=0.026) were found to be higher in the CPB-ALI group compared to the CPB-NALI group. The ratios of peripheral blood cells parameters before and after surgery (such as PostL/PreL, PostM/PreM, and PostN/PreN) showed no significant differences between two CPB groups. Comparison of clinical outcomes between CHD infants after CPB with ALI and those without ALI revealed that CPB-ALI infants had a longer ventilator time (median 67, IQR 24.5-10.05 vs. median 23, IQR 8.5-29, *p*=0.002) and a longer CCU stay (median 143, IQR 127-212.5 vs. median 72, IQR 68.5-149, *p*=0.006) compared to CPB-NALI patients.

**Table 1:** Demographic and clinical characteristics of three groups.

Characteristics	CON (n=13)	CPB-ALI (n=13)	CPB-NALI (n=13)	*p*-value
Preoperative factors				
Age (months)	4.20 (3.80, 4.80)	3.13 (3.00, 3.40)	5.70 (2.30, 6.40)	0.172
Weight (Kg)	4.79 ± 1.02	5.931 ± 1.417	5.903 ± 1.710	0.077
Male (%)	53.8	53.8	46.2	1.000
Breast-feeding (%)	46.15	53.85	38.46	0.919
LVEF (%)	‐	65.76 ± 4.03	64.85 ± 3.44	0.539
PAH (%)	‐	76.9	53.8	0.411
SPO_2_ (%)	‐	98 (93.5, 100)	98 (87, 99)	0.333
RACHS-1≥2 (%)	‐	92.3	84.6	1.000
PreWBC count (×10^9^/L)	‐	8.6 (7.73, 10.80)	9.8 (9.27, 11.24)	0.144
PreHb (g/L)	‐	109 (101, 118.5)	120 (105.5, 138.5)	0.174
PrePlt count (×10^9^/L)	‐	265 (241.5, 367)	341 (299, 409)	0.069
PreL count (×10^9^/L)	‐	4.65 (2.82, 5.69)	6.34 (4.78, 7.03)	0.048
PreM count (×10^9^/L)	‐	0.7 (0.54, 1.33)	0.62 (0.53, 0.75)	0.228
PreN count (×10^9^/L)	‐	2.68 (1.23, 4.41)	2.67 (1.38, 3.29)	0.837
PreNL	‐	0.36 (0.20, 2.07)	0.36 (0.22, 0.55)	0.739
Operative factors				
CPB time (min)	‐	67.31 ± 19.28	65.23 ± 18.49	0.782
ACC time (min)	‐	37.38 ± 10.64	38.69 ± 12.70	0.778
Postoperative factors				
PostPaO_2_/FiO_2_	‐	1.06 (0.78,1.32)	2.80 (2.38,3.25)	<0.001
PostWBC count (×10^9^/L)	‐	11.95 (9.77, 18.45)	12.07 (7.52, 13.65)	0.522
PostPlt count (×10^9^/L)	‐	160 (129, 197.5)	222 (133, 264)	0.166
PostL count (×10^9^/L)	‐	1.47 (1.15, 1.84)	2.15 (1.51, 2.76)	0.026
PostM count (×10^9^/L)	‐	1.49 (0.99, 1.97)	0.85 (0.57, 1.11)	0.005
PostN count (×10^9^/L)	‐	8.86 (7.31, 13.57)	9.19 (4.54, 10.9)	0.369
PostNL	‐	5.66 (4.35, 8.06)	3.42 (1.49, 6.21)	0.026
PostL/PreL	‐	0.36 (0.26, 0.46)	0.32 (0.23, 0.52)	0.261
PostM/PreM	‐	1.91(1.50, 2.72)	1.40 (0.95, 1.92)	0.598
PostN/PreN	‐	4.67 (1.81, 7.55)	3.67 (0.96, 6.68)	0.319
Clinical Outcomes				
VT (hours)	‐	67 (24.5, 10.05)	23 (8.5, 29)	0.002
CCU time (hours)	‐	143 (127, 212.5)	72 (68.5, 149)	0.006
VT ≥ 3 days (%)	‐	46.2	0	0.015

RACHS-1, Risk Adjustment in Congenital Heart Surgery-1; LVEF, Left Ventricular Ejection Fractions; PAH, Pulmonary hypertension; SPO_2_, oxygen saturation; PreWBC, Preoperative white blood cells; PreHb, Preoperative hemoglobins; PrePlt, Preoperative platelets; PreN, Preoperative neutrophils; PreLC, Preoperative lymphocyte; PreM, Preoperative monocytes; PreNL, Preoperative neutrophil-to-lymphocyte; CPB, Cardiopulmonary bypass; ACC, Aortic cross-clamping; PostPaO_2_/FiO_2_, postoperative ratio of partial pressure of O_2_ in arterial blood to fraction of inspired oxygen on the first postoperative day; PostWBC, Postoprative white blood cells; PostPlt, Postoprative platelets; PostN, Postoprative neutrophils; PostL, Postoprative lymphocytes; PostM, Postoprative monocytes; PostNL, Postoprative neutrophil-to-lymphocyte on the first postoperative day; VT, Ventilator time; CCU, Coronary Care Unit.

### 3.2 Microbiota dysbiosis signatures differ in the CPB-ALI compared to CPB-NALI and CON

**Figure 1:**
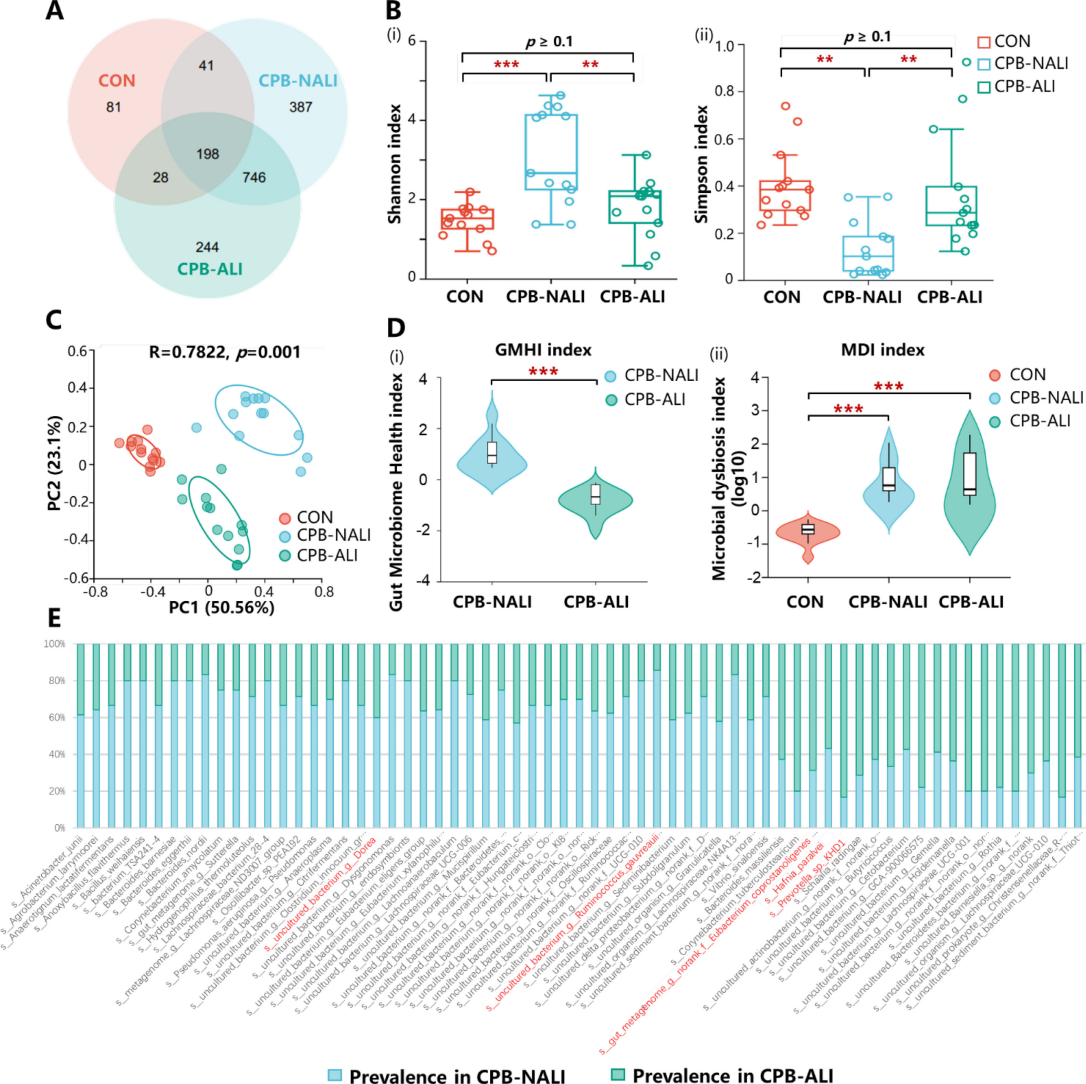
Microbial profile among CHD infants with CPB-ALI, CPB-NALI and healthy groups. **(A)** A Venn diagram demonstrating the existence of OTUs in each group. **(B)** Beta diversity analysis based on PCoA plot. Each symbol represents the gut microbiota of a sample. **(C)** Significant difference in alpha diversity between CPB-ALI and non-ALI groups estimated by Shannon (i) and Simpson (ii) index. **(D)** Significant difference in GMHI (i) and MDI (ii) between CPB-ALI and non-ALI groups. **(E)** Species prevalence in the respective groups of CPB-ALI and CPB-NALI. Box-plot features represent the median (central line), upper and lower quartiles (box), and the maximum and minimum values of the data (bars). GMHI, Gut Microbiome Health Index; MDI, Microbial Dysbiosis Index. **p<0.01, and ***p<0.001.

Fecal samples obtained from all enrolled patients were analyzed using an Illumina NovaSeq platform for sequencing. The Sobs and Shannon rarefaction curves gradually flattened out, indicating a reasonable number of individual samples (**Supplementary Figure 1**). In all samples, a total of 1725 OUTs (97% identity) were observed. The Venn diagram displayed the common and unique OTUs detected in the CPB-ALI, CPB-NALI, and CON groups, respectively. Among them, the CON group, CPB-ALI group, and CPB-NALI group each had unique OTUs of 81, 244, and 387, respectively. There were 198 shared OTUs among the three groups, with 944 shared between CPB-ALI and CPB-NALI, and 226 shared between CPB-ALI and CON (**Figure 1A**). These results suggest that infants with CHD exhibited significantly greater diversity in their gut microbiota compared to individuals in good health, and infants without ALI after surgery had a richer microbiota than those with ALI. The microbial variety present in fecal samples was assessed by comparing the alpha-/beta- diversity. There were significant differences in alpha-diversity between the CPB-ALI and CPB-NALI groups, as indicated by the Shannon index (pWilcoxon = 0.0008) and Simpson index (pWilcoxon = 0.0003) (**Figure 1B**). Furthermore, based on PCoA evaluation of OTU, the β-diversity of microbiota structure exhibited significant differences among groups according to the weighted UniFrac (R = 0.7759, *p* = 0.001) (**Figure 1C**).

GMHI and MDI are robust index based on species-level taxonomic features of gut microbiome samples to assess health status, focusing on determining the likelihood of disease independently of clinical diagnoses; this is achieved by comparing the relative abundances of two microbial species associated with good and poor health status. In comparison to alpha indices, GMHI significantly outperforms ecological indices in distinguishing between healthy and unhealthy groups, and demonstrates strong reproducibility in stratification of healthy and unhealthy groups, often considered a marker of gut health and dysbiosis (30). In our study, we found that the GMHI values in the CPB-NALI group were significantly higher than those in the CPB-ALI group (1.127 ± 0.640 vs. -0.670 ± 0.466, *p*< 0.001) (**Figure 1D**i, **Supplementary Table 1**), while the MDI values were much lower than those in the CPB-ALI group (-0.699 ± 0.356 vs. 0.904 ± 0.445, *p*< 0.001) (**Figure 1D**ii, **Supplementary Table 1**).

In addition, we analyzed the distribution of prevalent species in the CPB-NALI and CPB-ALI groups (**Figure 1E**), counted the number and frequency of endemic and scarce species in each group, and calculated the absolute difference multiples of the prevalence of the same species in the two groups. Among the endemic species, *Eubacterium coprostanoligenes* and *Dorea* had high prevalence rate in both groups, reaching 92.31% in the CPB-NALI group, and 69.23% and 61.54% in the CPB-ALI group, respectively. The fold-change between the two groups were 1.3 and 1.5, respectively; The species has the largest fold-change between the two groups was *Ruminococcus gauvreauii*, with a 6-fold difference in prevalence rates, being 46.15% in the CPB-NALI group and 7.69% in the CPB-ALI group. In the scarce species, *Hafnia paralvei* had the highest prevalence in both groups, reaching 76.92% in the CPB-NALI group and 100% in the CPB-ALI group, respectively, with a difference of 1.3 times between the two groups; the species with the largest difference in prevalence between the two groups was *Prevotella*, with a 5-fold difference, prevalence of 7.69% in CPB-NALI and 38.46% in CPB-ALI (**Supplementary Table 2**). Moreover, the species composition within individual samples (**Supplementary Figure 2**) and different sets in Venn diagrams (**Supplementary Figure 3**) were also analyzed. All these findings indicate a significant dysbiosis in the gut microbiota of CPB-ALI compared to healthy controls and CPB-NALI.

### 3.3 Specific taxonomic signatures in CPB-ALI patients

**Figure 2:**
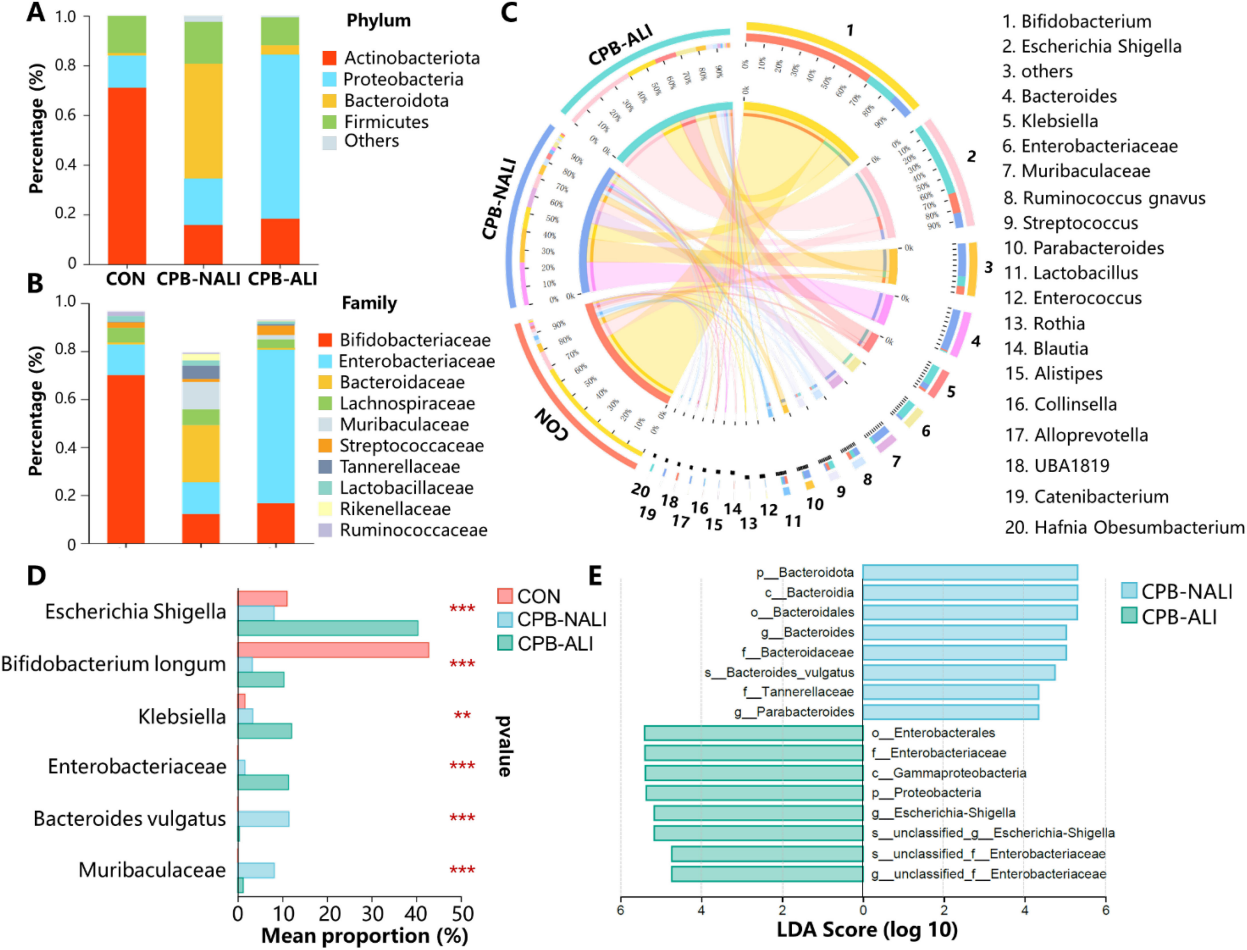
Taxonomic features of healthy, CPB-NALI and CPB-NALI groups. Relative abundances of bacteria between groups at the phylum **(A)**, family **(B)**, and genus **(C)** level. **(D)** Microorganisms with significant differences between the three groups were analyzed at the species level. Data were analyzed by ordinary one-way ANOVA with *post-hoc* Tukey **p<0.01, and ***p<0.001. **(E)** LEfSe used to identify essential differences in bacterial abundance (genus to species level) between the CPB-ALI and CPB-NALI groups. Only taxa with a significant LDA threshold value > 4 are shown.

We observed and analyzed the intestinal microbiota profiles in each group and found significant differences in the distribution of microbiota at various levels among the three groups. In all three groups, Actinobacteria, Proteobacteria, Bacteroidetes, and Firmicutes were the most common phyla at the phylum level. Firmicutes had similar abundance across the groups, while the other three phyla showed significant variations. In CHD infants who underwent CPB, there was a significant decrease in the abundance of Actinobacteriota compared to the healthy group (71.07% in CON, 18.25% in CPB-ALI, and 15.81% in CPB-NALI). Moreover, when comparing the two CPB groups, we noticed a rise in the presence of Proteobacteria (66.15%) in infants with ALI, while the abundance of Bactroidota increased (46.25%) in infants without ALI (**Figure 2A**, **Supplementary Figure 4**). CPB-ALI exhibited clear dysbiosis, with a significant increase in the abundance of Proteobacteriaceae, which are known for producing endotoxins and are considered the main pathogenic bacteria. As the abundance of Proteobacteria increases, it causes an imbalance between pathogenic bacteria and helpful symbiotic bacteria. Adherent and invasive Proteobacteria may take advantage of genetic abnormalities in recognizing pathogens and removing bacteria, leading to inflammation and an imbalance between the microbiota and the immune system in the mucous membranes. On the other hand, Bactroidota has the ability to adapt to host and intestinal environmental stress and possesses significant nutritional flexibility. The effect of Enterocella on the host is difficult to determine as it can have both negative and positive impacts. Bacteroides can be advantageous to the host by safeguarding against potential pathogens that might invade and cause infection, and it also stimulate the intestinal lining to produce fucosylglycans and promote angiogenesis in neonatal epithelium, which enhances nutrient absorption in the body.

In terms of the family level, when comparing the CPB-ALI group with the non-ALI group, it was found that Enterobacteriaceae had a higher presence and Bacteroidaceae had a lower presence (**Figure 2B**). A chart illustrating the connections between microbiota composition and the three groups was created at the genus level, and the genera that appeared the most frequently in all samples were Bifidobacterium, Escherichia-Shigella, Bacteroides, Klebsiella, and Enterobacteriaceae (**Figure 2C**). The control group exhibited an enrichment of *Bifidobacterium* (70.03%), while *Bacteroides* dominated in the CPB-NALI group (23.84%). In contrast, the main flora in CPB-ALI were *Escherichia-Shigella* (40.42%), with *Bacteroides* accounting for only 0.69% (**Supplementary Figure 4**). *Escherichia-Shigella* is recognized as an opportunistic pathogen that promotes inflammation onset and exacerbates disease progression (31). *Bacteroides* is the primary producer of short-chain fatty acids in the human intestine, and its metabolites, mainly acetic acid and propionic acid, act as effective anti-inflammatory mediators (32). These metabolites can inhibit the release of pro-inflammatory cytokines by neutrophils and macrophages, helping to maintain intestinal homeostasis and immune system stability. Overall, our findings suggest that CPB-ALI may be associated with a decrease in certain probiotic microbe taxa and an increase in opportunistic pathogens.

The abundance of the top 6 different species was analyzed among the CON, CPB-ALI, and CPB-NALI groups using one-way analysis of variance. It is important to note that *Bifidobacterium longum* was the dominant bacteria in the CON group. In comparison, the CPB-ALI group had a higher enrichment of *Escherichia Shigella* sp., *Klebsiella* sp., and *Enterococcus* sp., which are common pro-inflammatory opportunistic pathogens, compared to the CPB-NALI group. Additionally, the abundance of *Bacteroides vulgatus* was higher in the CPB-NALI group (**Figure 2D**, **Supplementary Table 3**). Furthermore, a LefSe analysis was conducted to identify the dominant bacterial species in the CON, CPB-ALI, and CPB-NALI groups. The results revealed that CPB-ALI showed a higher enrichment of *Escherichia Shigella*, *Klebsiella*, and *Enterococcus*, while *Bacteroides vulgatus* was more abundant in the CPB-NALI group (**Figure 2E**, **Supplementary Table 4**).

### 3.4 Relationships between functional pathways, inflammatory indicators, and gut microbiota in CPB-ALI

**Figure 3:**
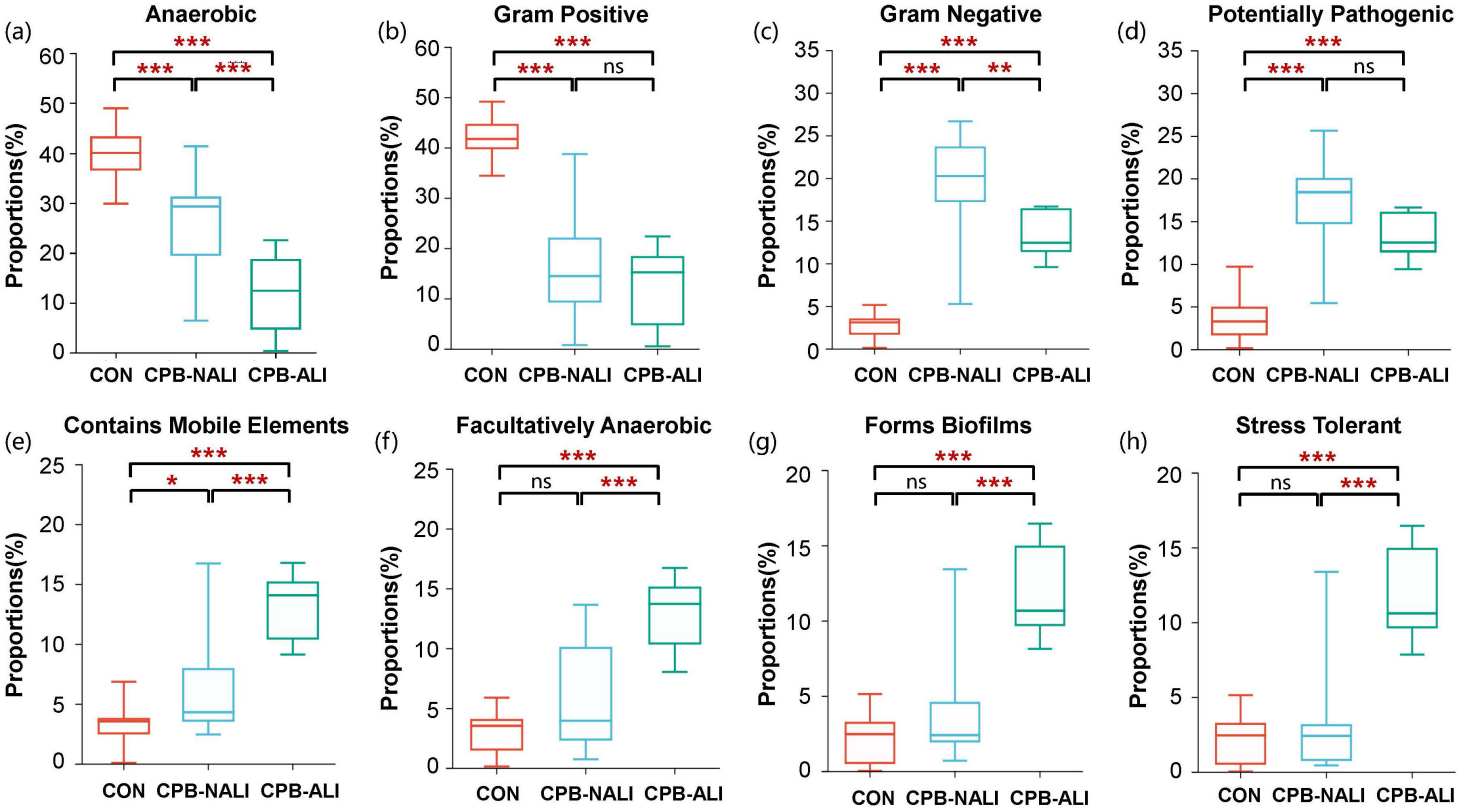
The bacterial phenotype function in CON, CPB-NALI, and CPB-ALI groups are based on BugBase predictive analysis, including anaerobic **(A)**, Gram-positive **(B)**, Gram-negative **(C)**, pathogenic potential **(D)**, presence of mobile elements **(E)**, oxygen utilization **(F)**, biofilm formation **(G)**, and oxidative stress tolerance **(H)**. *p < 0.05, **p < 0.01, and ***p < 0.001.

BugBase analysis was performed to predict the bacterial phenotypes and functions in the CON, CPB-NALI, and CPB-ALI groups. All eight potential phenotypes are shown in **Figure 3**. Compared to the CON group, both CPB groups had significantly higher relative abundance of gram-negative bacteria that are potentially pathogenic and contain mobile elements. They also had significantly lower abundance of anaerobic and gram-positive bacteria (*p*<0.001). While the two CPB groups did not show significant differences in the abundance of Gram-positive bacteria and potential pathogens (**Figures 3B, D**), they exhibited significant differences in the other six potential phenotypes. CPB-ALI had a significantly higher abundance of mobile elements, facultatively anaerobic bacteria, bacteria that form biofilms, and stress-tolerant bacteria compared to CPB-NALI (**Figures 3E–H**). On the other hand, CPB-ALI had a significantly lower abundance of anaerobic and gram-negative bacteria (**Figures 3A, C**). Notably, CPB-ALI showed the highest abundance of facultatively anaerobic bacteria, bacteria that form biofilms, and stress-tolerant bacteria among all three groups (*p*<0.001), while there was no significant difference observed between CON and CPB-NALI (**Figures 3F–H**). This suggests that facultatively anaerobic bacteria, which form biofilms and exhibit stress tolerance, could potentially be associated with the development of postoperative complications in CPB-ALI.

**Figure 4:**
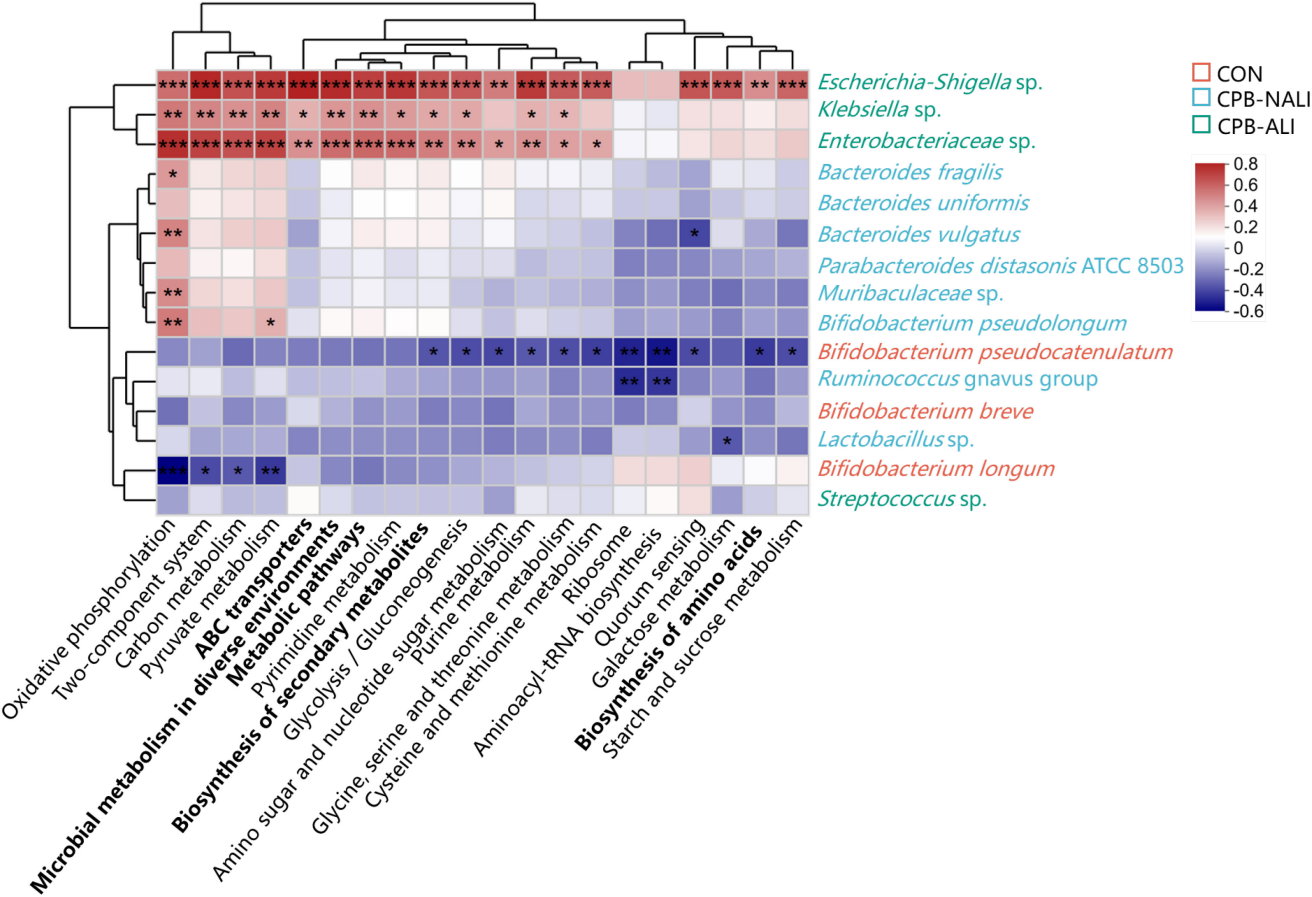
Heat map of the correlations between top 20 KEGG pathway and gut microbiota at the species level. Columns: Pathways predicted by PICRUSt2 among the three groups, and the top 5 abundance pathways were highlighted in bold; Rows: Differential species. Species highlighted in red, green and blue were enriched in the CON group, the CPB-ALI group and the CPB-NALI group, respectively. *p < 0.05, **p < 0.01, and ***p < 0.001. Heatmap scale values represent correlation coefficients, and the positive and negative numbers indicating positive and negative correlations, respectively. A larger absolute value signifies a stronger correlation.

Moreover, distinct pathways with specific functions were anticipated within the CON, CPB-ALI, and CPB-NALI groups. PICRUSt2 was used to analyze the 16S rDNA sequencing data at the third level, resulting in a mapping of 909 KEGG modules. The abundance of *Escherichia Shigella*, *Klebsiella*, and *Enterobacteriacea* in CPB-ALI patients was linked to various pathways related to energy metabolism, signal transduction, global and overview maps, carbohydrate metabolism, membrane transport, nucleotide metabolism, amino acid metabolism, translation, and cellular community – prokaryotes (**Supplementary Table 5**). Specifically, the metabolic pathways, biosynthesis of secondary metabolites, microbial metabolism in diverse environments, biosynthesis of amino acids, and ABC transporters showed significant differences between CPB-ALI and CPB-NALI (**Supplementary Figure 5**, **Supplementary Table 5**). Compared to the CPB-ALI fecal samples, the dominant bacteria in the CPB-NALI group, such as *Bacteroides fragilis*, *Bacteroides vulgatus*, and *Bifidobacterium pseudolongum*, had a positive correlation with oxidative phosphorylation, while *Bacteroides vulgatus* had a negative correlation with quorum sensing. In the CON group, the dominant bacteria *Bifidobacterium pseudocatenulatum* and *Bifidobacterium longum* had a negative correlation with all the top 20 pathways with the highest abundance (**Figure 4**, **Supplementary Table 5**).

**Figure 5:**
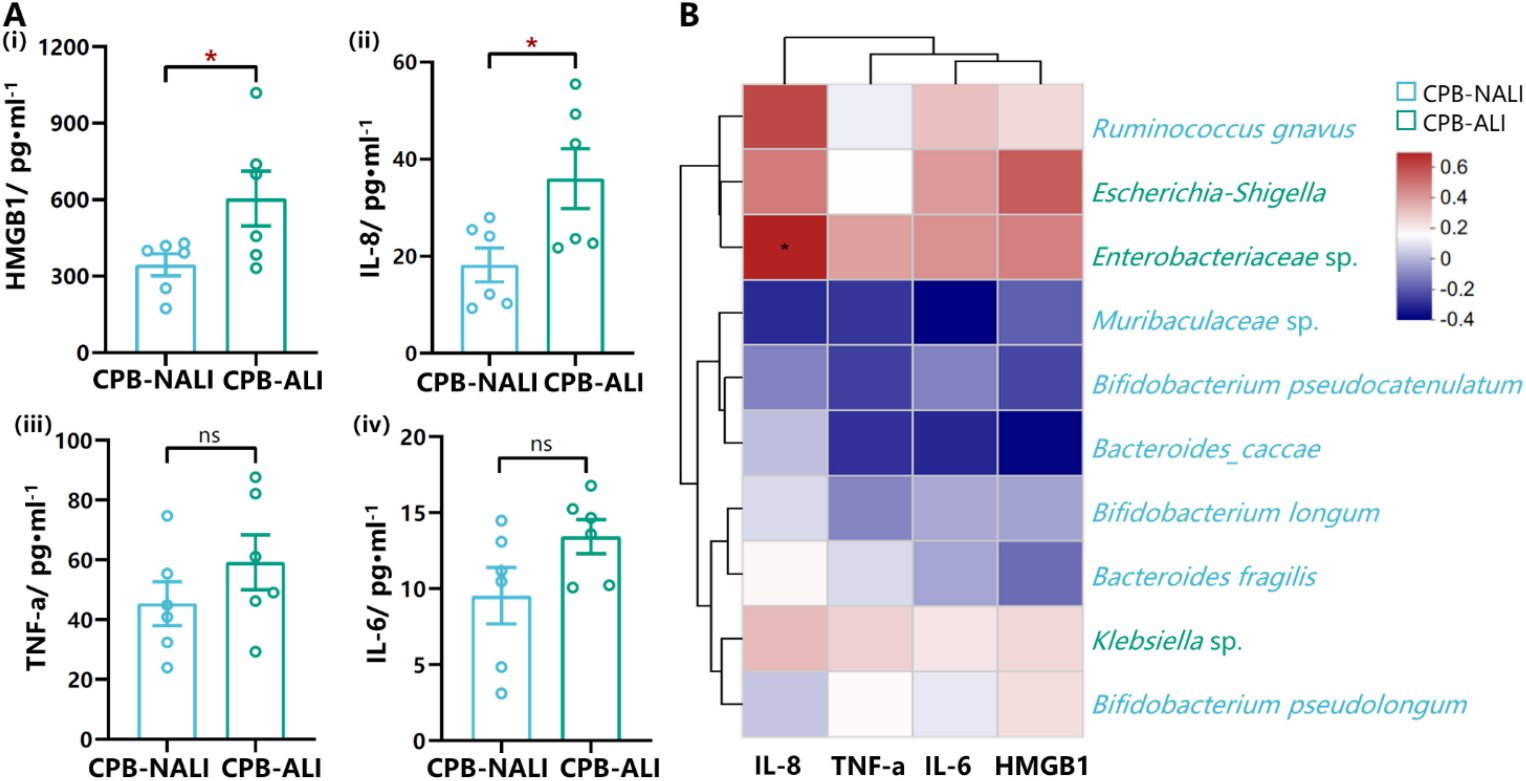
Levels of cytokines from serum in CPB-ALI and CPB-NALI groups and their correlations with the microbiota. **(A)** The levels of HMGB1 (i), IL-8 (ii), TNF-a (iii) and IL-6 (iv) in serum from CPB-ALI and CPB-NALI groups were detected by ELISA. Each dot represented an individual. Statistical analysis was performed using *t* test analysis, *p < 0.05. **(B)** Spearman’s correlation heatmap showing the associations of between the TOP 10 species in gut microbiota and inflammatory cytokines. Different colors indicate correlation level; *p <0.05. Heatmap scale values represent correlation coefficients, and the positive and negative numbers indicating positive and negative correlations, respectively. A larger absolute value signifies a stronger correlation.

HMGB1 (High mobility group box-1 protein) is an important factor involved in mediating infection, tissue damage, and inflammation, and is present at extremely low levels in the serum of healthy individuals. It has been reported that under conditions of intestinal ischemia-reperfusion, HMGB1 can aggravate postoperative ALI occurrence by mediating neutrophil formation of extracellular traps (33). Immune cells release various cytokines after being stimulated by HMGB1, such as macrophages increasing the synthesis of TNF and the expression level of TNF mRNA; monocytes releasing TNF, IL-1α, IL-1β, IL-6, and IL-8; and neutrophils increasing the secretion of TNF, IL-1, and IL-8 (34).Therefore, we examined the levels of HMGB1, IL-8, TNF-α, and IL-6 in the serum of patients before surgery. Data shows that levels of HMGB1 and IL-8 in the preoperative serum of CPB-NALI patients were significantly lower than those in the CPB-ALI group (*p*<0.05) (**Figure 5A**i,ii). Meanwhile, levels of TNF-a and IL-6 in the serum of patients in both groups were similar (*p*>0.05) (**Figures 5A**iii,iv). In addition, a heatmap analysis further examined the relationship between microbiota and levels of these four inflammatory factors, showing a significant correlation between IL-8 and Enterobacteriaceae in the gut microbiota (**Figure 5B**). The above results suggest that an increase in pathogenic bacteria leads to microbiota dysbiosis in CPB-ALI patients, and activates HMGB1 as the inflammatory “alarm”, further participating in the upregulation of IL-8 to mediate subsequent inflammatory responses.

### 3.5 The gut microbiota *Escherichia Shigella* showed a correlated with clinical indicators

**Figure 6:**
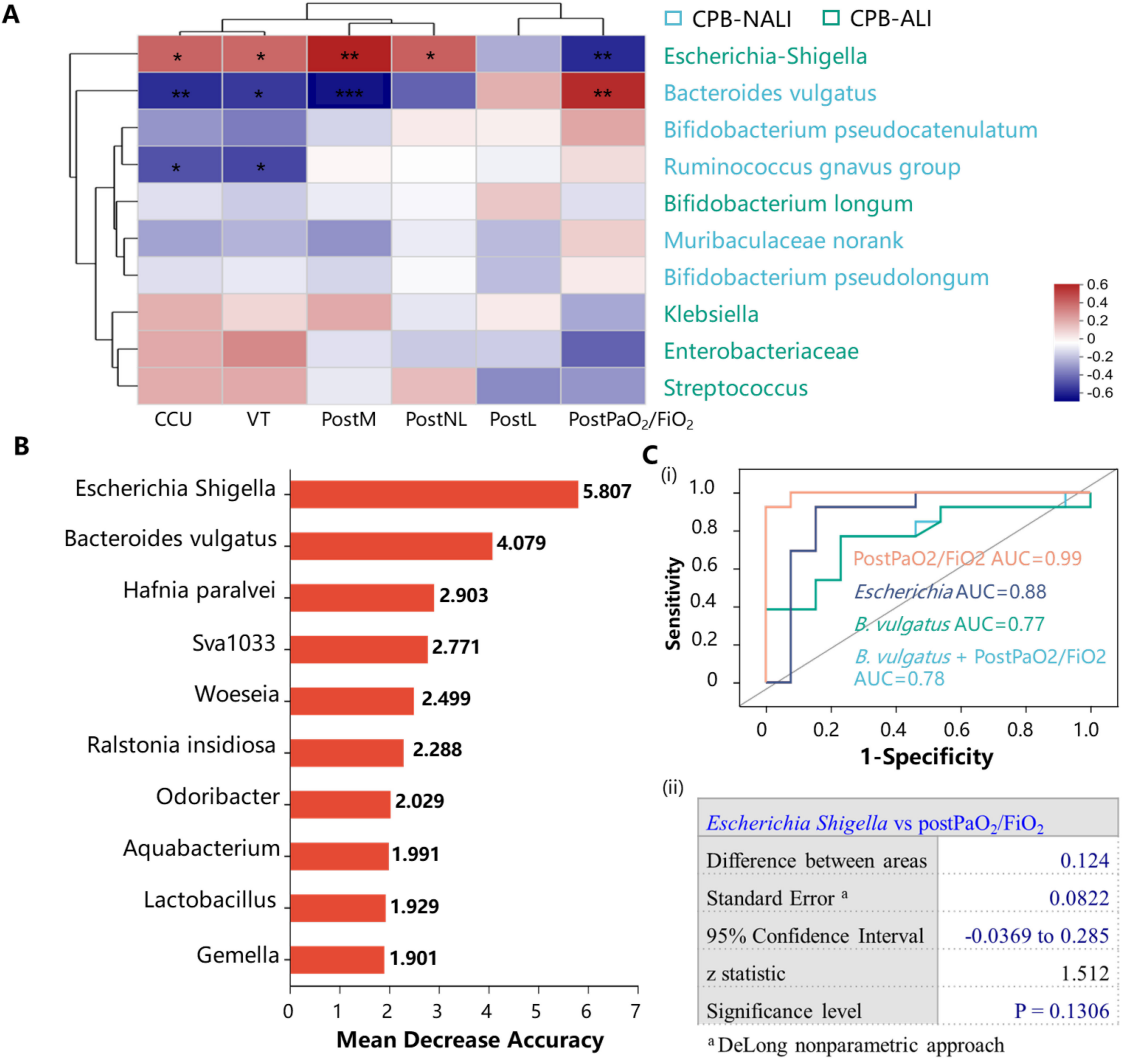
Bacterial taxonomic biomarkers to predict CPB-ALI. **(A)** Associations of specific microbiota in different levels with clinical characteristics. The heatmap of Spearman’s rank correlation coefficients between the gut microbiota and clinical indicators. *p <0.05, **p <0.01, and ***p <0.001; VT, Ventilator time; PostPaO_2_/FiO_2_, postoperative oxygenation index after CPB, heatmap scale values represent correlation coefficients, and a larger absolute value signifies a stronger correlation; **(B)** The top 10 biomarker bacterial classes were identified by applying random forests regression to their relative abundance values. Potential biomarker taxa are ranked in descending order of importance to the accuracy of the model. The inserted figure shows the 10-fold cross-validation error as a function of the number of species used to regress against the CPB-ALI in order of variable importance; **(C)** ROC curves showed the ability of specific microbiome biomarker in predicting CPB-ALI (i), and no significant difference in AUC between *Escherichia Shigella* as a biomarker and postoperative oxygenation index (ii). ROC, receive operating characteristic; AUC, area under ROC curve; a, DeLong nonparametric approach.

We performed a Spearman correlation analysis to investigate the relationship between gut microbiota and various clinical indicators, such as demographic characteristics, laboratory test, severity of symptoms, and outcomes. We found that the differences in gut microbiota may contribute to these clinical phenomena. *Escherichia Shigella* showed higher abundance in CPB-ALI compared to CPB-NALI and exhibited moderate to strong positive correlations with CCU stay, ventilator time, PostM, PostNL, and disease severity indicated by PostPaO_2_/FiO_2_ (PaO_2_/FiO_2_ is the ratio of arterial partial pressure of oxygen (PaO_2_) to inspired oxygen concentration (FiO_2_), known as the oxygenation index, which is used to assess a patient’s respiratory function and oxygenation status). Conversely, all these clinical symptoms showed an inverse correlation with the abundance of Bacteroides vulgatus, which was more abundant in the CPB-NALI group. *Ruminococcus gnavus*, which had a higher abundance in CPB-NALI, showed a moderate negative correlation with CCU stay and ventilator time (**Figure 6A**).

To investigate whether *Escherichia Shigella*, *B. vulgatus*, or other gut microbial signatures could predict CPB-ALI, we used random forest to detect specific bacteria linked to poor outcomes, as depicted in **Figure 6B**. *Escherichia Shigella* exhibited the best predictive capacity as a biomarker for CPB-ALI. ROC curve analysis revealed that the predictive capability of the presence of *Escherichia Shigella* (AUC 0.88, 95% CI: 0.71–1.00) was superior to that of *B. vulgatus* (AUC 0.77, 95% CI: 0.57–0.96) in terms of diagnosing a certain condition. There was no significant difference in AUC between *Escherichia Shigella* as a biomarker and the postoperative oxygenation index (AUC 0.99, 95% CI: 0.98–1.00) in the context of CPB-ALI (*p* = 0.7104), indicating the considerable predictive potential of *Escherichia Shigella* (**Figure 6C**).

## 4 Discussion

In this study, we investigated the composition of the gut microbiota in patients with CPB-ALI, CPB-NALI, and healthy controls. Our aim was to explore the correlation between gut microbiota and clinical parameters and outcomes in CHD patients, with the goal of identifying a non-invasive biomarker for potential CPB-ALI infants. Our findings revealed significant differences in gut microbial composition and diversity between the two groups, as well as differences in microbial community distribution at various levels. Specifically, we found that a specific species called Escherichia-Shigella was more abundant in the CPB-ALI group compared to the CPB-NALI group. We discovered that the relative abundance of Escherichia-Shigella in the preoperative gut of infants positively correlated with VT, CCU stay, and oxygenation index after surgery. Furthermore, *Escherichia-Shigella* exhibited high accuracy (AUC=0.88) in predicting the occurrence of CPB-ALI in ROC analysis. Additionally, we found that *B. vulgatus* in CPB-NALI patients was significantly negatively correlated with multiple indicators and clinical outcomes after surgery, suggesting a potential protective role in CPB-injured infants. These findings highlight the potential of gut microbiota changes and probiotic regulation as intervention measures for lung injury.

The lungs and intestines share the same embryonic origin, resulting in similarities in their physiological structure. Numerous studies have demonstrated the important regulatory role of the gut microbiota and its metabolic by-products. For example, there is a positive correlation between serum succinic acid levels following adult cardiac surgery and markers of lung injury, mechanical ventilation time, and a negative correlation with oxygenation index. This indicates a link between post-CPB lung injury and gut ischemia-reperfusion injury (8). One proposed mechanism involves succinate, a metabolite produced by the intestinal microbiota, which polarizes alveolar macrophages through the SUCNR1-dependent pathway, exacerbating ALI induced by intestinal ischemia-reperfusion (9). Additionally, HMGB1 has been shown to mediate neutrophil formation of extracellular traps, thereby worsening acute lung injury caused by intestinal ischemia-reperfusion (33). Our study is the first to present the correlation between postoperative lung injury outcomes and imbalances in intestinal microbiota in CHD infants undergoing CPB surgery. This provides clinical evidence and a theoretical basis for targeted research on postoperative lung injury in infants.

In our study, CHD infants have more diverse microbiota than healthy. This could be due to the underlying disease states in congenital heart disease patients, which might disrupt the natural balance of their microbiota, potentially increasing its diversity. This increase in diversity may also indicate dysbiosis, where the typical intestinal microbial balance is altered. Stress and inflammation associated with ALI can lead to decreased microbial diversity, with pathogenic bacteria dominating and beneficial bacteria diminishing. Inflammatory cytokines and other immune mediators released during ALI can alter the gut environment, favoring the growth of opportunistic pathogens like *Shigella* while suppressing beneficial bacteria such as *Bacteroides*. This shift results in an overall reduction in microbial diversity. Interestingly, the alpha diversity index, which measures diversity within samples, is similar between healthy controls and ALI infants. This similarity may arise because, despite differing health statuses, both groups might share a core set of microbial species necessary for maintaining baseline diversity. However, the composition and function of these communities may differ significantly, with ALI infants harboring more pathogens and fewer beneficial microbes.

The opportunistic pathogen Escherichia-Shigella, with pro-inflammatory potential, dominates the gut of patients with CPB-ALI, with Klebsiella and Enterobacter comprising 40.42% and 12.11%, respectively. It is speculated that the abundance of these pathogenic bacteria promotes the secretion of inflammatory factors, disrupts gut microbiota homeostasis, and further exacerbates inflammation, ultimately leading to poor postoperative outcomes in CPB-ALI patients. Similar findings have been observed in various other studies, such as a significant elevation in the relative abundance of *Escherichia-Shigella* in the gut of patients with non-alcoholic fatty liver disease (35), tuberculous meningitis (36), or Alzheimer’s disease (37), along with positive correlations with pro-inflammatory cytokines and chemokines (37, 38). These research results strongly support our findings. The genus Bacteroides constitutes approximately 30% of the human intestinal microbiota and plays a crucial role in the colonic ecosystem (32). In our study, we observed enrichment of *Bacteroides vulgatus* in the intestines of CPB-NALI patients, accounting for 11.5% of the total bacterial community. *B. vulgatus* is known for its ability to produce succinate and propionate (39) and possesses an enzyme system for degrading complex polysaccharides (40). It is also involved in the synthesis of probiotics and bioactive compounds (41), which are often associated with health benefits in humans and animals. Furthermore, studies have shown a significant decrease in the abundance of *B. vulgatus* and *B. dorei* in the gut of patients with coronary artery disease (42). Animal experiments have demonstrated that increasing the abundance of these two species can improve endotoxemia, reduce bacterial lipopolysaccharide production in the gut microbiota, and inhibit pro-inflammatory immune responses (43). These findings may contribute to the rapid recovery observed in CPB-NALI patients after surgery. However, there are also reports linking *B. vulgatus* to disease exacerbation, such as in patients with ulcerative colitis or polycystic ovary syndrome. In these cases, the extracellular abundance of *B. vulgatus* in the intestine is considered a key factor in damaging intestinal barrier function or affecting the secretion of beneficial metabolites in the gut (43, 44). Although *Bacteroides* is not the predominant species in infant gut microbiota (comprising only 0.87% in our healthy control group), increasing the abundance of *Bacteroides* appears to have a positive impact on neurodevelopment in late infancy (45).

The correlation between *Shigella* and ALI, and *Bacteroides* and non-ALI, suggests a possible causal relationship. Shigella is known for its pathogenicity and pro-inflammatory properties, which can exacerbate systemic inflammation and worsen the severity of ALI. In contrast, Bacteroides, which produces anti-inflammatory short-chain fatty acids (SCFAs), may help maintain gut balance and prevent inflammation, thereby reducing ALI risk. Shigella can worsen ALI prognosis through various mechanisms, including endotoxin production that triggers strong immune responses and increases pro-inflammatory cytokines like IL-8. This inflammatory cascade can compromise the gut barrier, allowing bacteria and endotoxins to enter the bloodstream, further aggravating lung injury. Additionally, Shigella disrupts the balance of the gut microbiota, decreasing beneficial bacteria that produce SCFAs and other anti-inflammatory compounds, impairing the communication between the gut and lung axes, and intensifying pulmonary inflammation and damage. Overall, the observed differences in microbial diversity and composition among infants with CON, ALI, and non-ALI groups highlight the complex interplay between the gut microbiota and host health. Further research, including longitudinal studies and functional analyses of the microbiota, is needed to elucidate the exact mechanisms of these associations and their impact on patient outcomes.

Overall, our study provides insights into the composition of the gut microbiota in CPB-ALI patients and its correlation with clinical parameters and outcomes. The findings suggest the potential use of gut microbiota as a non-invasive biomarker for identifying CPB-ALI infants and highlight the possibility of targeted interventions for postoperative lung injury in infants. However, there are some limitations in this study. Firstly, the sample size for each group was relatively small and therefore should be validated by a multicenter study. Secondly, gut microbiome samples were collected only at one time point upon admission, neglecting longitudinal surveillance studies with multi-site sampling. Finally, the use of metagenomic sequencing and multi-omics methods would allow for in-depth analysis and provide insights into multi-level interactions, enhancing our understanding of underlying mechanisms.

While the V4-V5 region of the 16S rRNA gene is commonly used due to its high variability and sufficient length to provide taxonomic resolution, it does have limitations in comparison to the full 16S gene sequencing. The full 16S gene provides a more comprehensive analysis as it includes nine hypervariable regions (V1-V9), offering a higher resolution and more accurate taxonomic identification, especially at lower taxonomic levels. Using the full 16S gene sequencing can mitigate potential biases introduced by focusing on specific regions, which might overlook certain microbial taxa or lead to misclassification. Furthermore, full-length 16S sequencing can enhance the detection of rare or novel organisms, thus providing a more holistic view of the microbial community. Therefore, while V4-V5 sequencing is cost-effective and sufficient for broad community profiling, the use of full 16S gene sequencing could yield more informative and reliable results, particularly in studies requiring precise microbial identification and diversity assessment.

Research conducted in the past decade has revealed the significant role of gut microbiota in human health. Generally, gut microbiota is beneficial to the host, particularly in terms of immunity and metabolism. However, an increasing number of studies have shown that gut microbiota is associated with disease processes and can serve as a biomarker for various diseases, including Type II diabetes, pancreatic cancer, colorectal cancer, heart failure, cirrhosis, and central nervous system diseases (46–50). Although this field of research is still relatively new, gut microbiota has demonstrated advantages such as non-invasiveness, high diagnostic efficiency, and accuracy, highlighting its potential as an important biomarker for early disease diagnosis. Ultimately, this may contribute to the development of more targeted intervention measures.

## 5 Conclusion

To the best of our knowledge, this is the initial study endeavor to investigate the characteristics of gut bacteria in infants with CHD who develop ALI after CPB, employing 16S rRNA gene sequencing technology. We revealed significant differences in the diversity of microbes, functional pathways, and composition of the gut microbiota among the groups, which were associated with poor clinical outcomes. Importantly, we identified *Escherichia-Shigella* as potential microbial biomarkers that might assist in predicting the development and outcome of CPB-ALI. This study offers new insights into the pathophysiological changes and underlying mechanisms involved in CPB-ALI, with important implications for identifying CHD infants at risk for developing ALI after CPB. Additional validation using multi-omics approaches, experimentation on animals, and trials involving human subjects are needed to verify our results and gain a better understanding of the underlying processes.

## Data Availability

The datasets presented in this study can be found in online repositories. The names of the repository/repositories and accession number(s) can be found in the article/**Supplementary Material.**
