# Supplementary figures and images for: Predicting acute lung injury in infants with congenital heart disease after cardiopulmonary bypass by gut microbiota
Source: Front Immunol. 2024 Oct 24;15:1362040. doi: 10.3389/fimmu.2024.1362040 (PMC11540645; doi:10.3389/fimmu.2024.1362040)

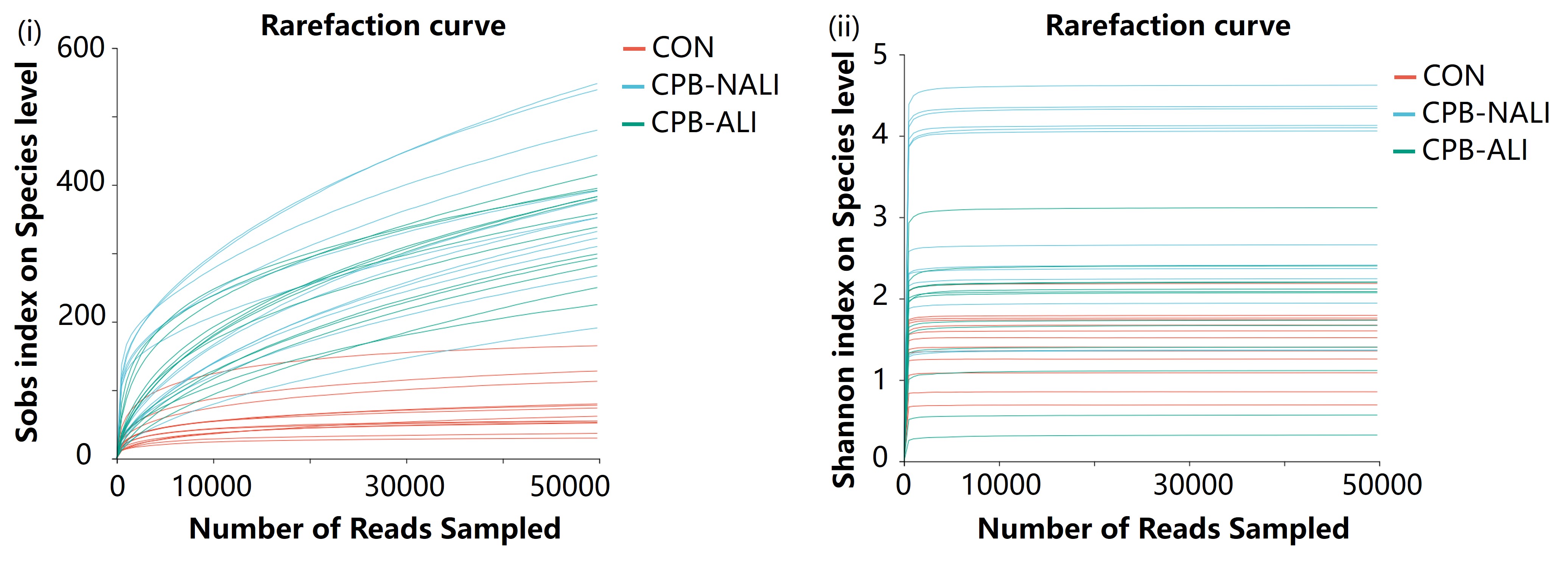

Supplement: Supplementary file 6 [file Image1.jpeg]

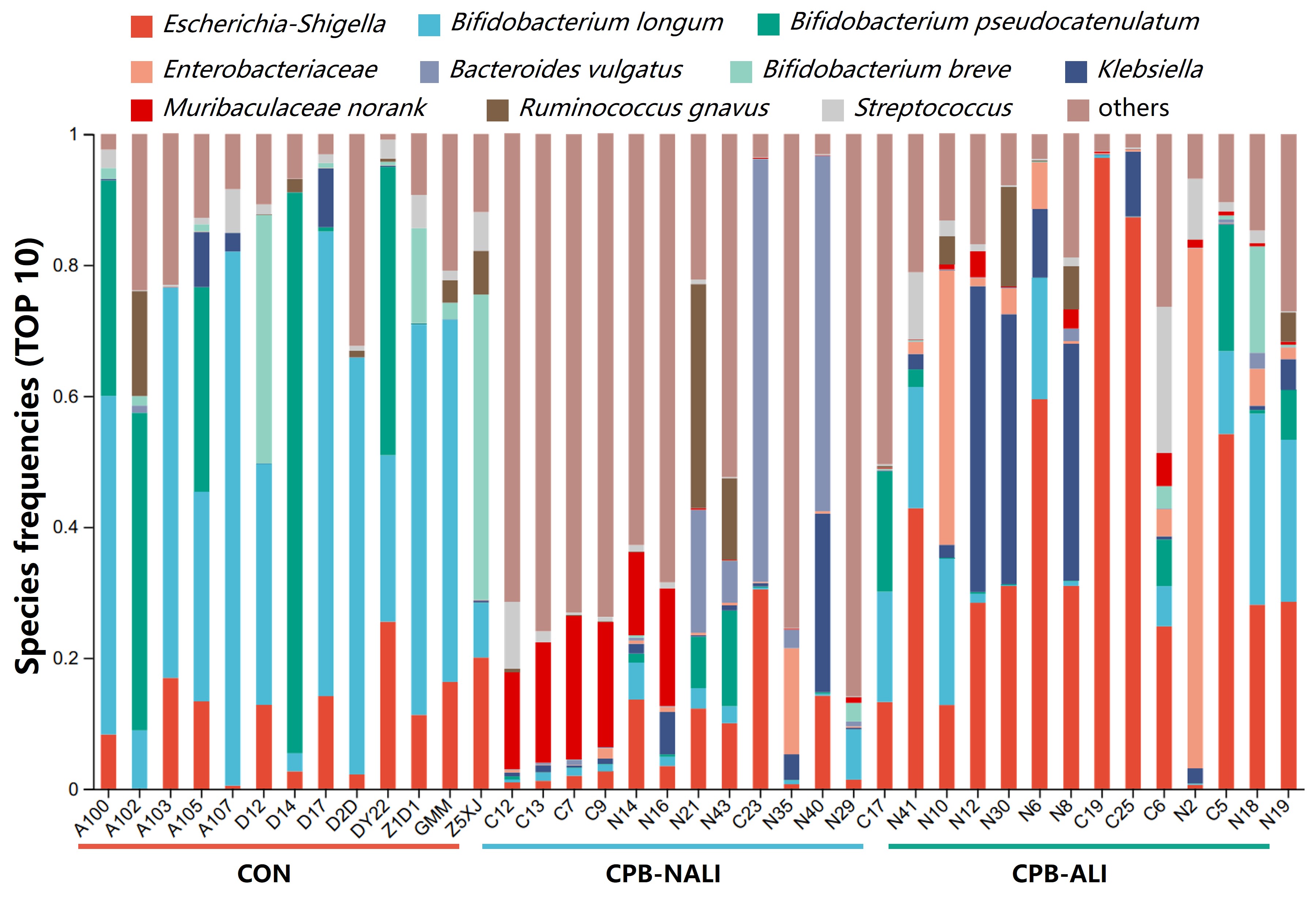

Supplement: Supplementary file 7 [file Image2.jpeg]

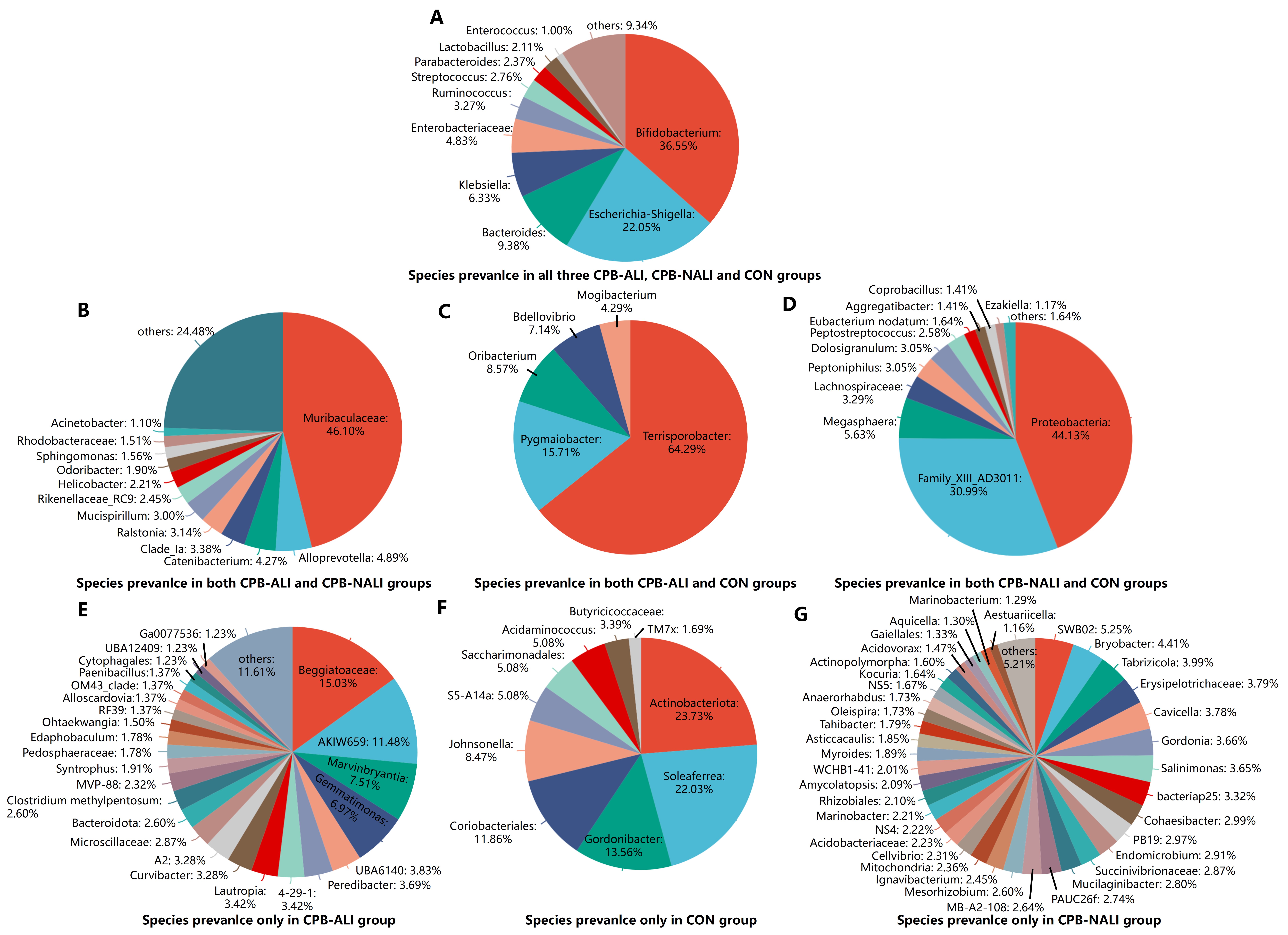

Supplement: Supplementary file 8 [file Image3.jpeg]

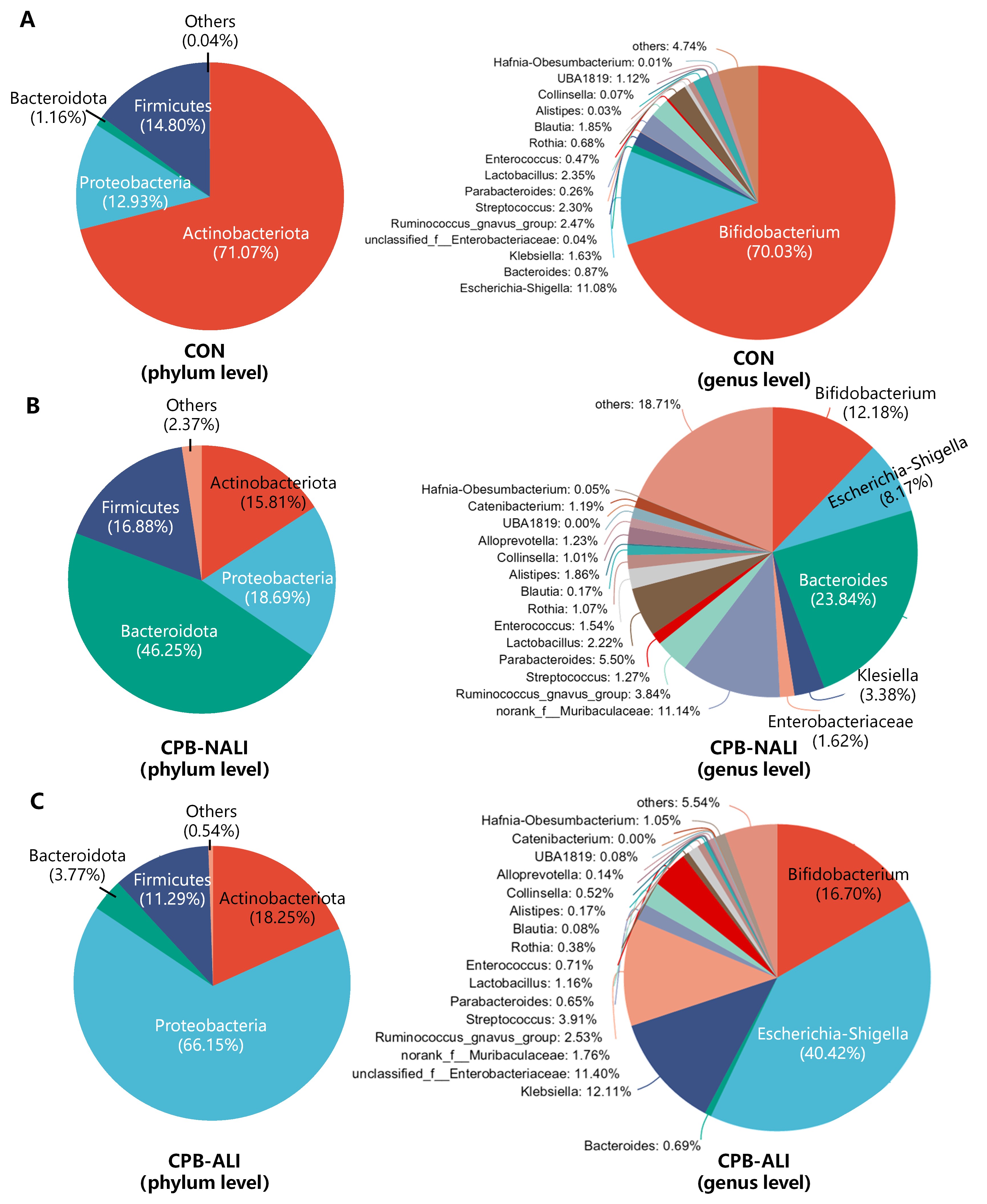

Supplement: Supplementary file 9 [file Image4.jpeg]

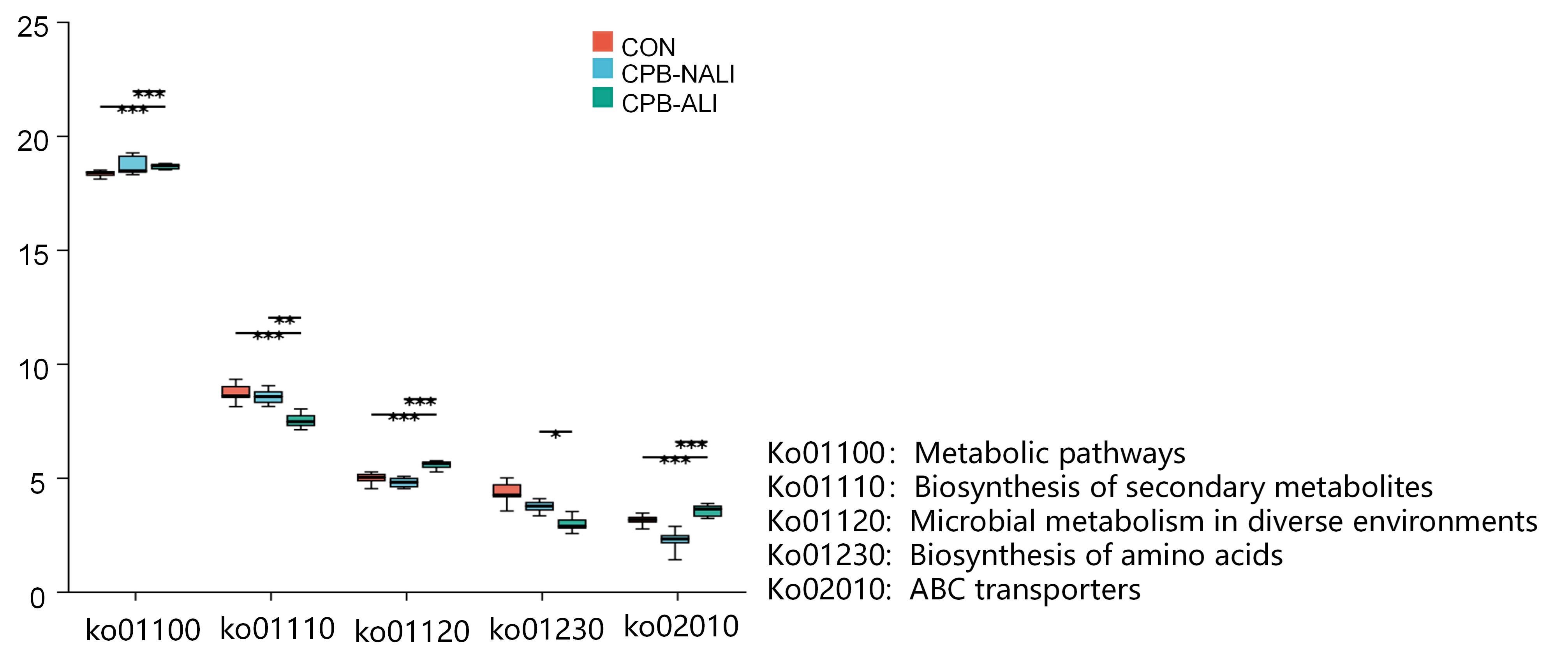

Supplement: Supplementary file 10 [file Image5.jpeg]
